# Supplementary material for: Enzyme-substrate hybrid β-sheet controls geometry and water access to the γ-secretase active site
Source: Commun Biol. 2023 Jun 24;6:670. doi: 10.1038/s42003-023-05039-y (PMC10290658; doi:10.1038/s42003-023-05039-y)
Supplement: Supplementary file 2 — Supporting Information [file 42003_2023_5039_MOESM2_ESM.pdf]

Supporting Information:

Enzyme-substrate hybrid β-sheet controls geometry and water access to the γ-secretase active site

Shu-Yu Chen[1], Lukas P. Feilen[2], Lucía Chávez-Gutiérrez[3,4], Harald Steiner[2,5], Martin Zacharias[1*]

[1]Center of Functional Protein Assemblies, Technical University of Munich, Garching, Germany
[2]German Center for Neurodegenerative Diseases (DZNE), Munich, Germany
[3]VIB-KU Leuven Center for Brain & Disease Research, Leuven, Belgium
[4]Department of Neurosciences, Leuven Research Institute for Neuroscience and Disease (LIND), KU Leuven, Leuven, Belgium
[5]Biomedical Center (BMC), Division of Metabolic Biochemistry, Faculty of Medicine, LMU Munich, Germany

* To whom correspondence should be addressed: zacharias@tum.de

Supplementary Figure S1: Illustration of the distance-based criteria for a catalytically active geometry in the Aβ52-bound D385^{H} -γ-secretase.

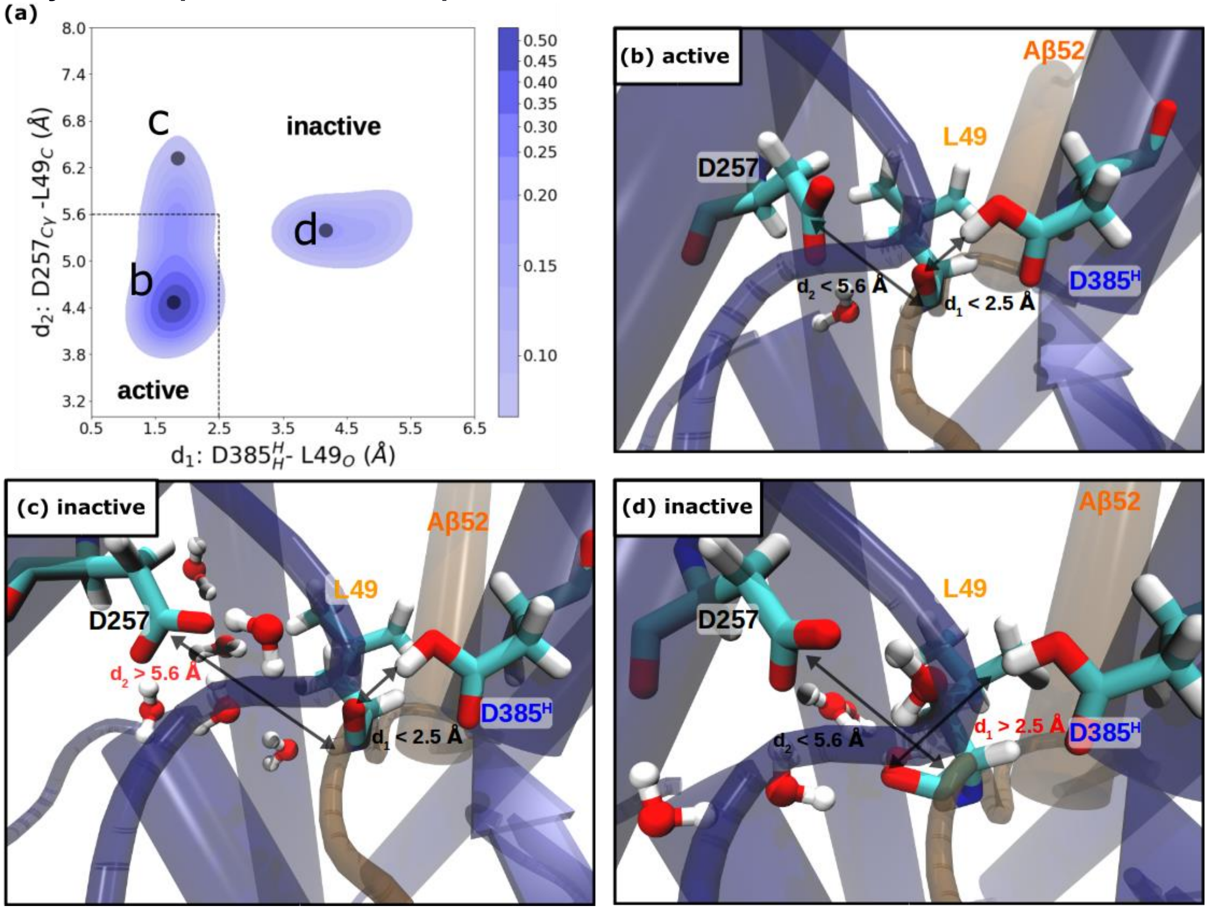

(a) 2D distribution contour map with the distance of the catalytic hydrogen bond (d_1) as x-axis and vs. the distance between the substrate carbonyl and the Cγ of the deprotonated aspartate (d_2) in a 600ns trajectory. Black dots indicate the examples of one active geometry and two inactive geometries. The 2 dashed lines represent the 2 distance-based criteria d_1 =2.5Å and d_2 =5.6Å and separate the active conformations (lower left) from the inactive states (the rest). The color scale represents the distribution density. **(b)** Conformation of an active geometry fulfilling both distance-based criteria. **(c)** Conformation of an inactive geometry with a large d_2 (> 5.6Å) filled with more than one water molecules. **(d)** Conformation of an inactive geometry without the catalytic hydrogen bond (d_1 < 2.5Å).

Supplementary Figure S2: Detailed geometries of the post-cleavage site region

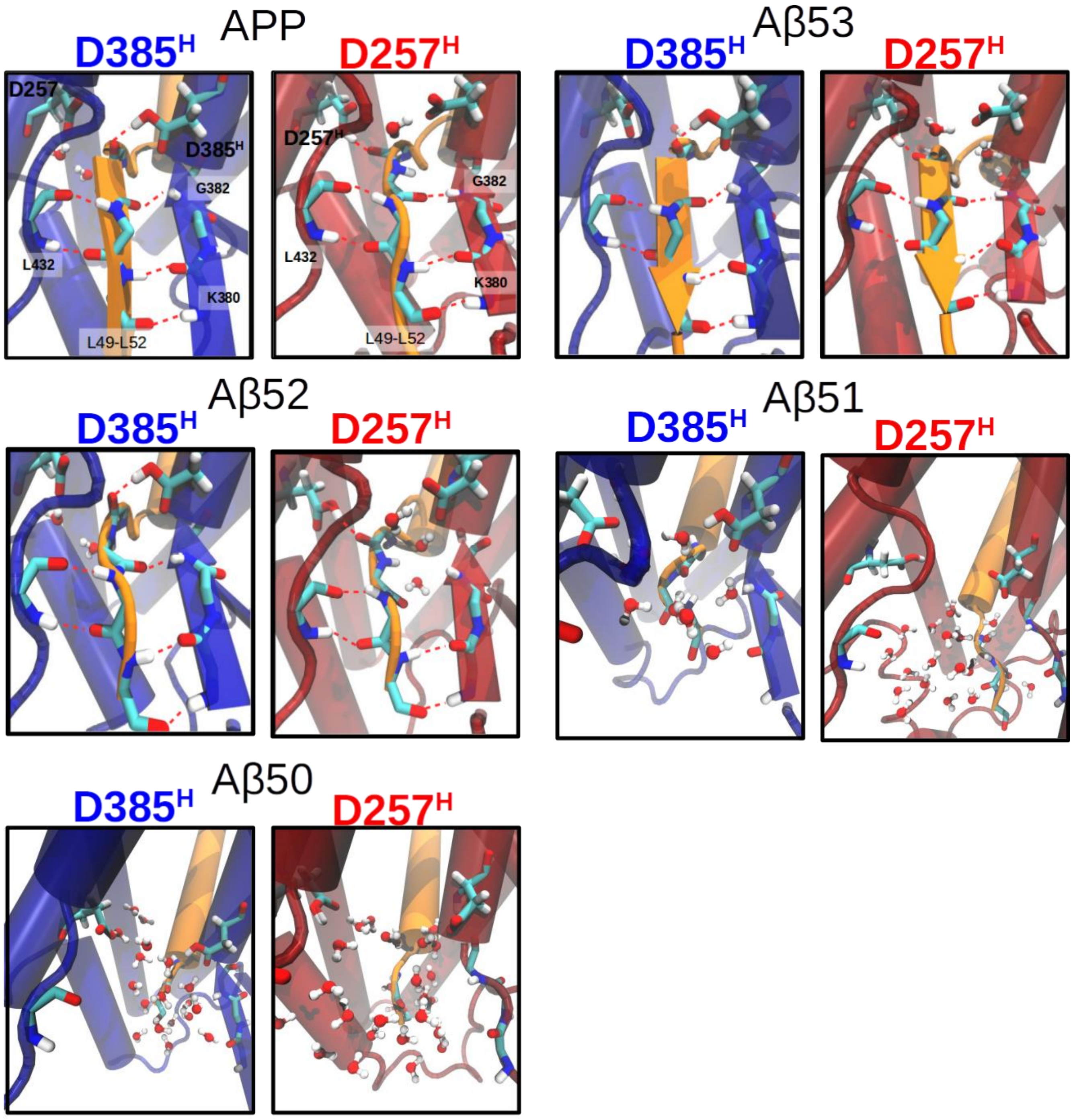

Detailed structure at the post-cleavage site region of γ-secretase in complex with APP and its truncated variants. In each enzyme-substrate complex, $D385^{H}$-PS1 is shown in blue in the left panel, $D257^{H}$-PS1 is shown in red in the right panel, and substrates are shown in orange. PS1 D257, D385 and backbone atoms involved in the hydrogen network are shown in licorice representation with substrate-PS1 hydrogen bonds (red dashed lines) including: L49-D257/D385, V50-G382, M51-L432, and L52-K380. Water within 5Å of L49-L52 of the substrate are shown in stick-ball representation.

Supplementary Figure S3: Detailed features statistics of the unrestraint MD simulations of APP and its truncated variants.

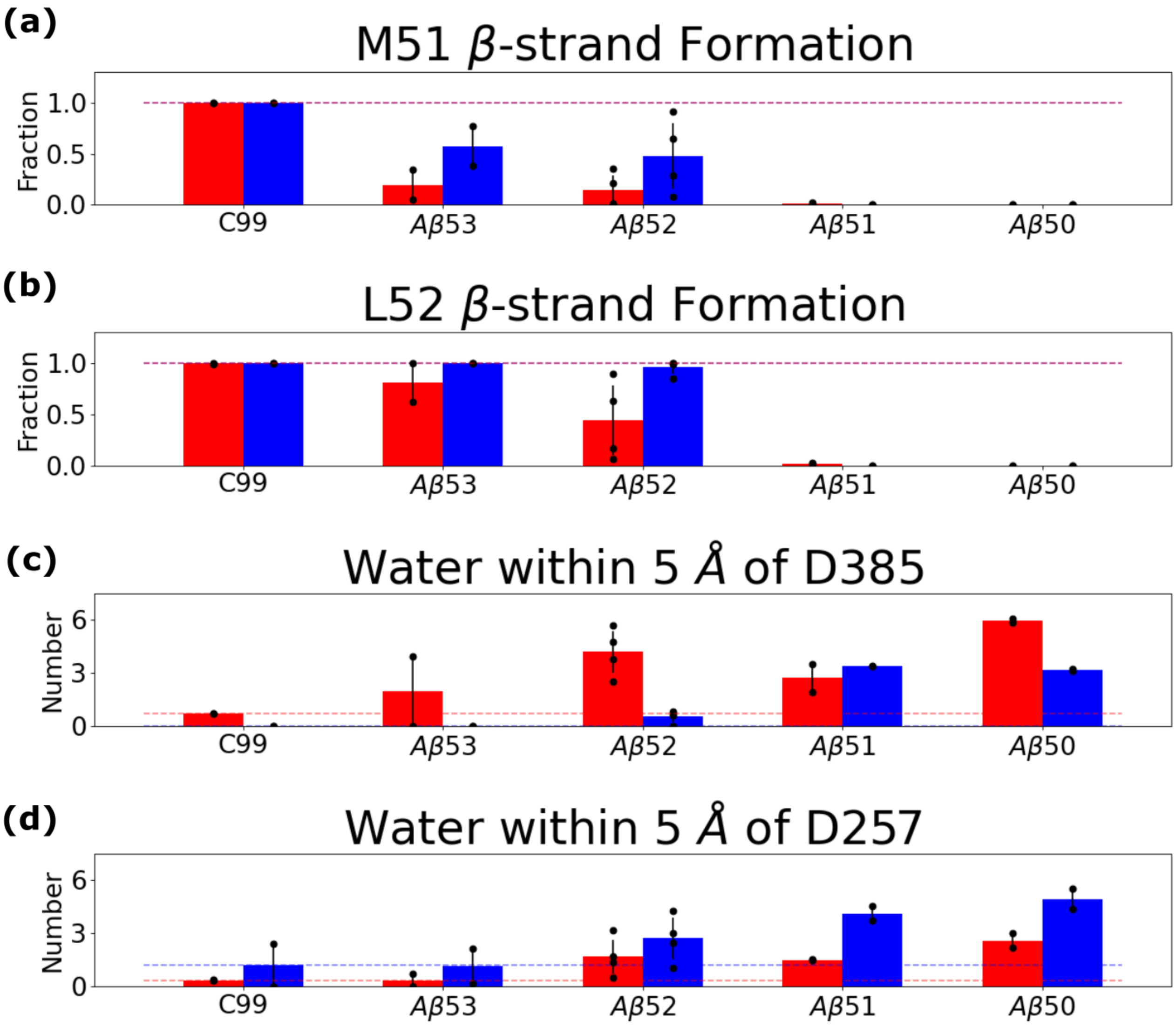

(a) β-strand occupation fraction on M51. **(b)** β-strand occupation fraction on L52. **(c)** Averaged water counts within 5Å of D385 of PS1. **(d)** Averaged water counts within 5Å of D257 of PS1. The blue and red dashed lines show the average values measured in WT $D385^{H}$-PS1 and $D257^{H}$-PS1, respectively. Error bars show the standard deviation of the mean of each system ($n \geq 2$).

Supplementary Figure S4: **Secondary structures over time calculated by DSSP method in APP and its truncated variants binding to D385^{H} γ-secretase.**

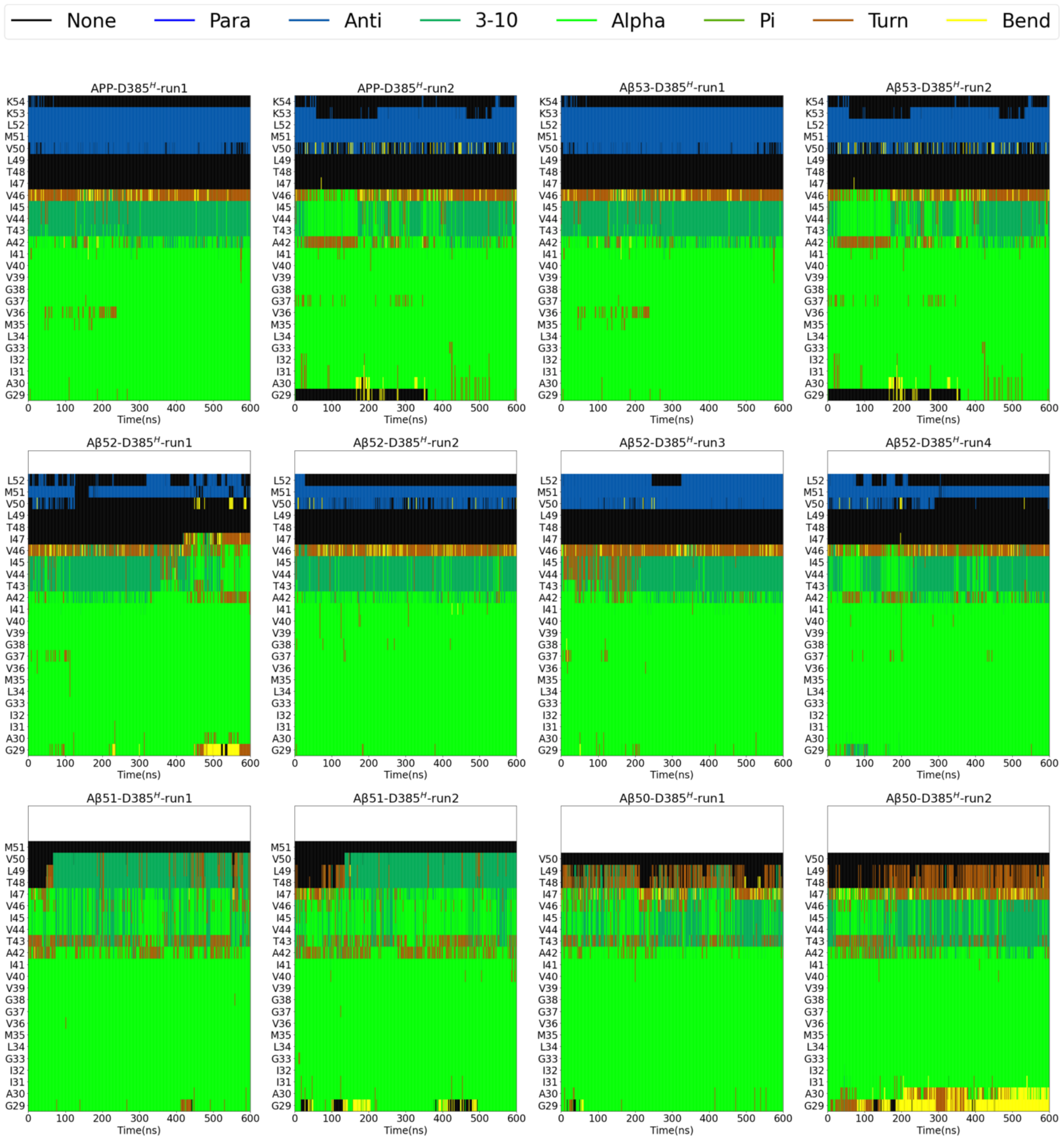

Time evolution of the secondary structure of the substrate TMDs analyzed using the DSSP method.

Supplementary Figure S5: Secondary structures over time calculated by DSSP method in APP and its truncated variants binding to D257^{H} γ-secretase.

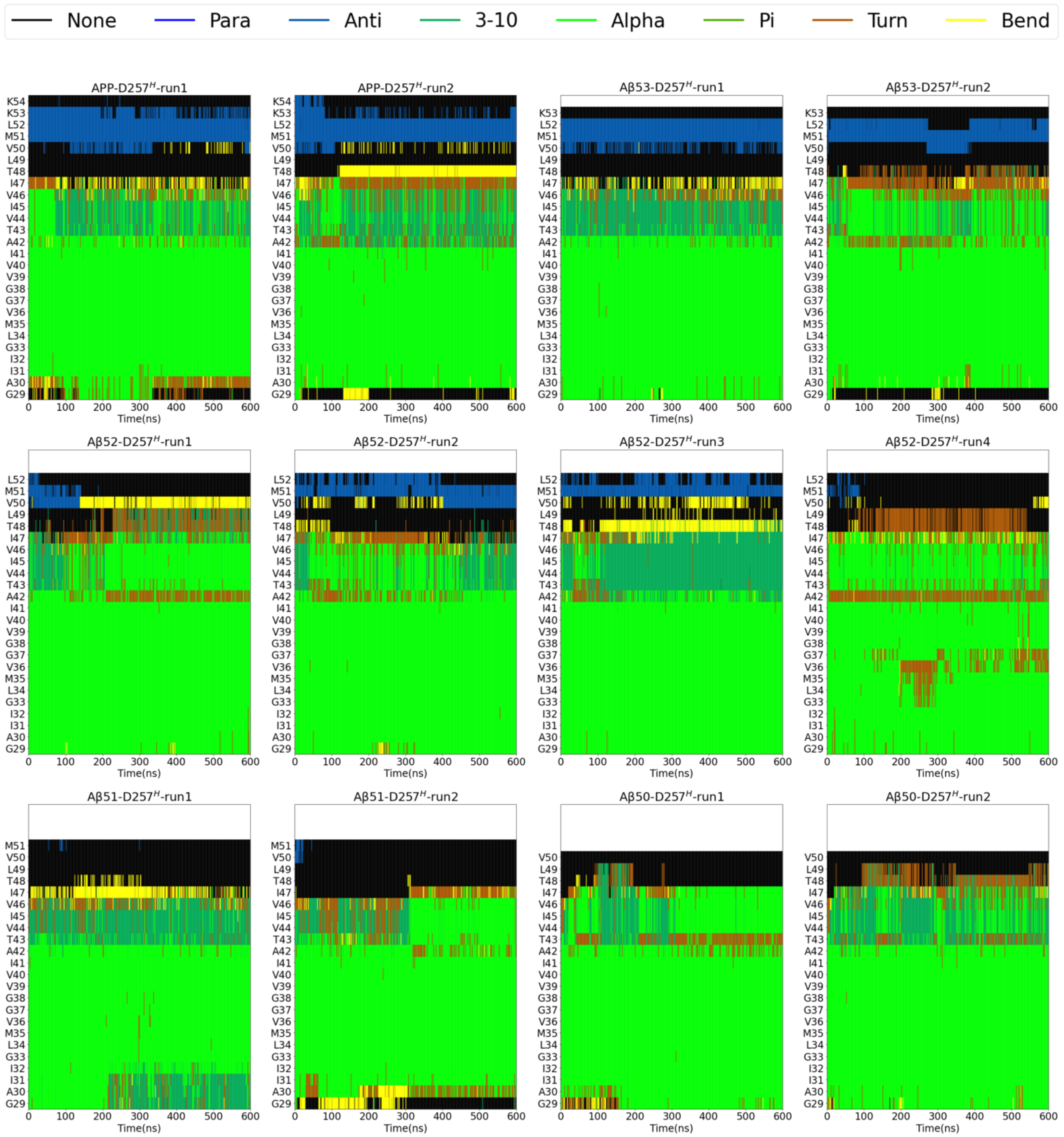

Time evolution of the secondary structure of the substrate TMDs analyzed using the DSSP method.

Supplementary Figure S6: Water residence time around the catalytic center.

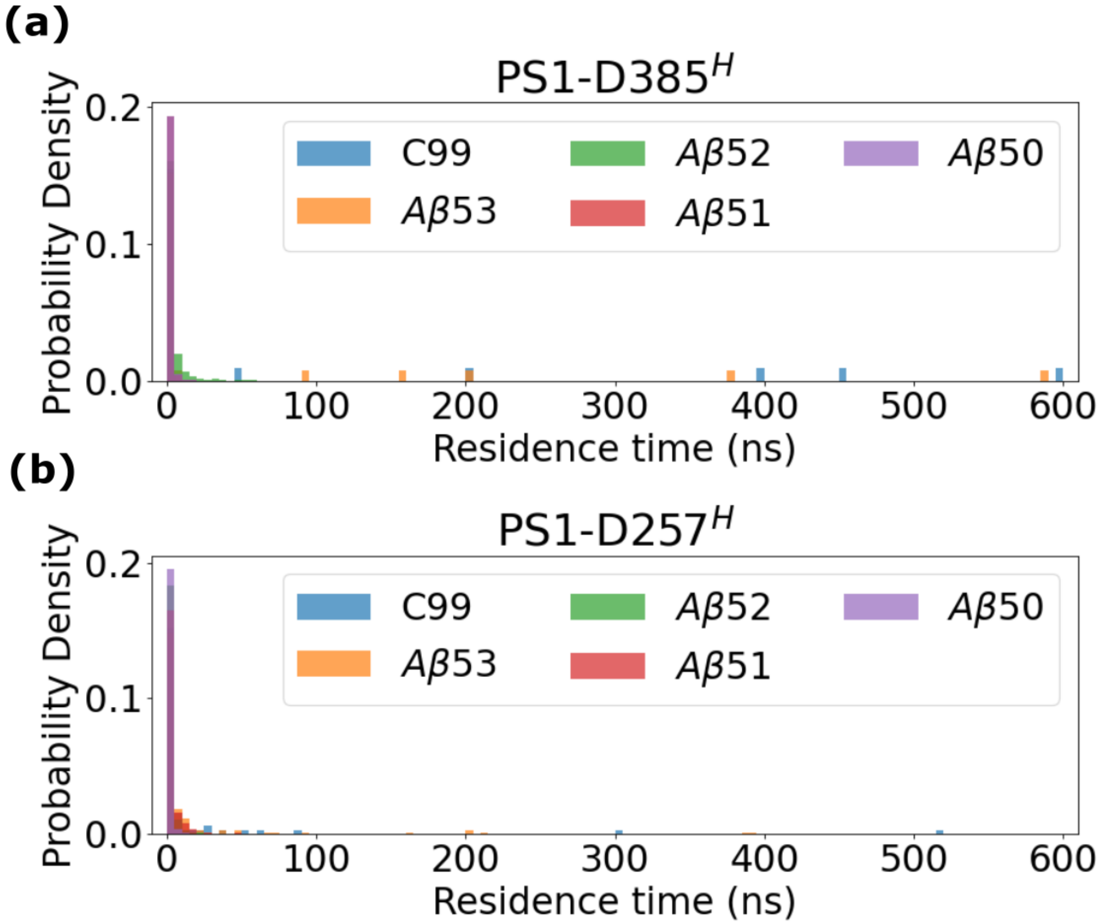

(a) Distribution of water residence time around the catalytic center when substrate binds to γ-secretase-PS1-D385^{H}. **(b)** Distribution of water residence time around the catalytic center when substrate binds to γ-secretase-PS1-D257^{H}. The data points are binned into intervals with a width of 5ns.

Supplementary Figure S7: Correlation between active geometry formation, β3-strand formation fraction, and number of water molecules around the catalytic center.

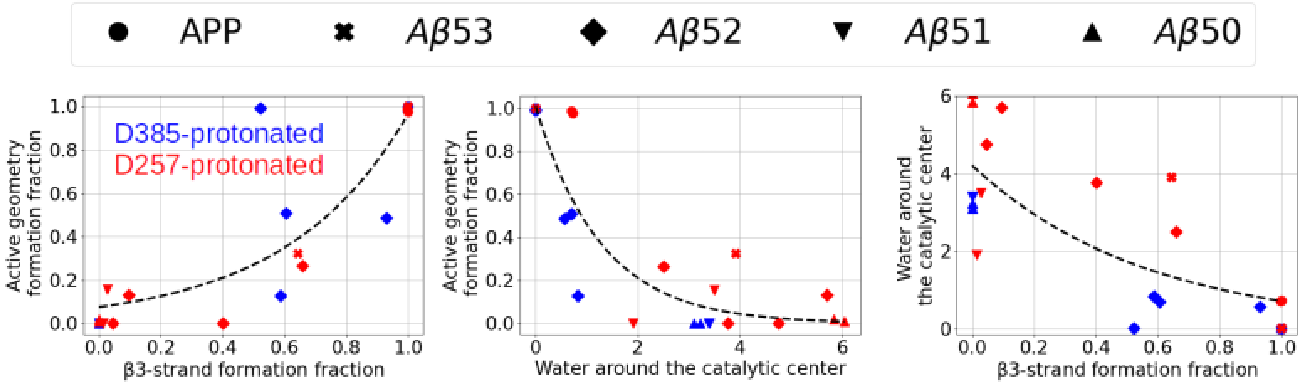

Data of each individual unrestrained MD simulations are shown with different marks indicating different substrates binding to γ-secretase-PS1-D385^{H} (blue) or γ-secretase-PS1-D257^{H} (red).

Supplementary Figure S8: MD simulations of γ-secretase complexes with APP and truncated substrates.

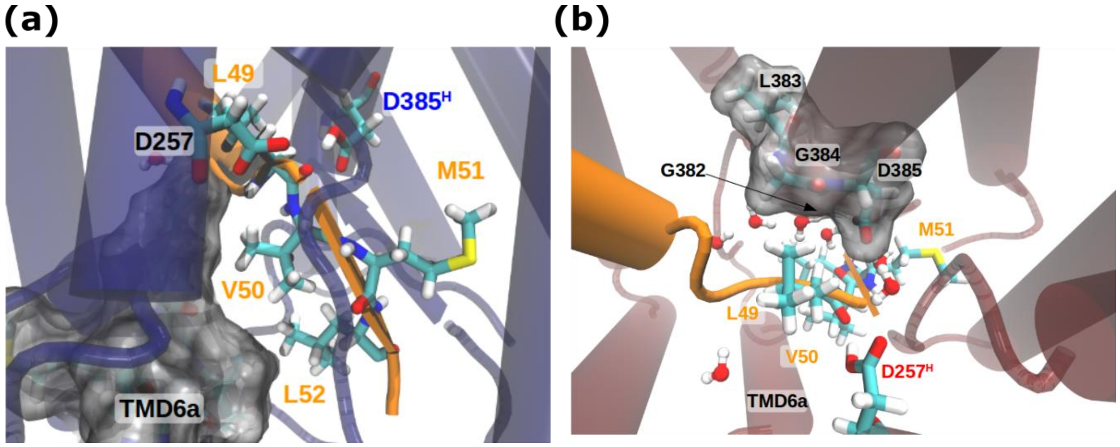

(a) A simulation snapshot illustrates hydrophobic contacts between substrate residues V50 (P2') and L52 (P3') of substrate Aβ53 and TMD6a of PS1-D385^{H}. The transparently white surface represents the Van der Waals surface of PS1 TMD6a (L268-R278). **(b)** Snapshot of the water-dwelling cavity formed by the GxGD motif in D257-protonated PS1. D257 and D385 of PS1 and substrate P1-P3' are shown in the licorice representation. Waters within 5Å of D257 or D385 are shown in ball-stick representation. The white surface represents the Van der Waals surface of GxGD motif on PS1 TM7 (G382-D385).

Supplementary Figure S9: Time-course of the data collected in each unrestraint MD simulation.

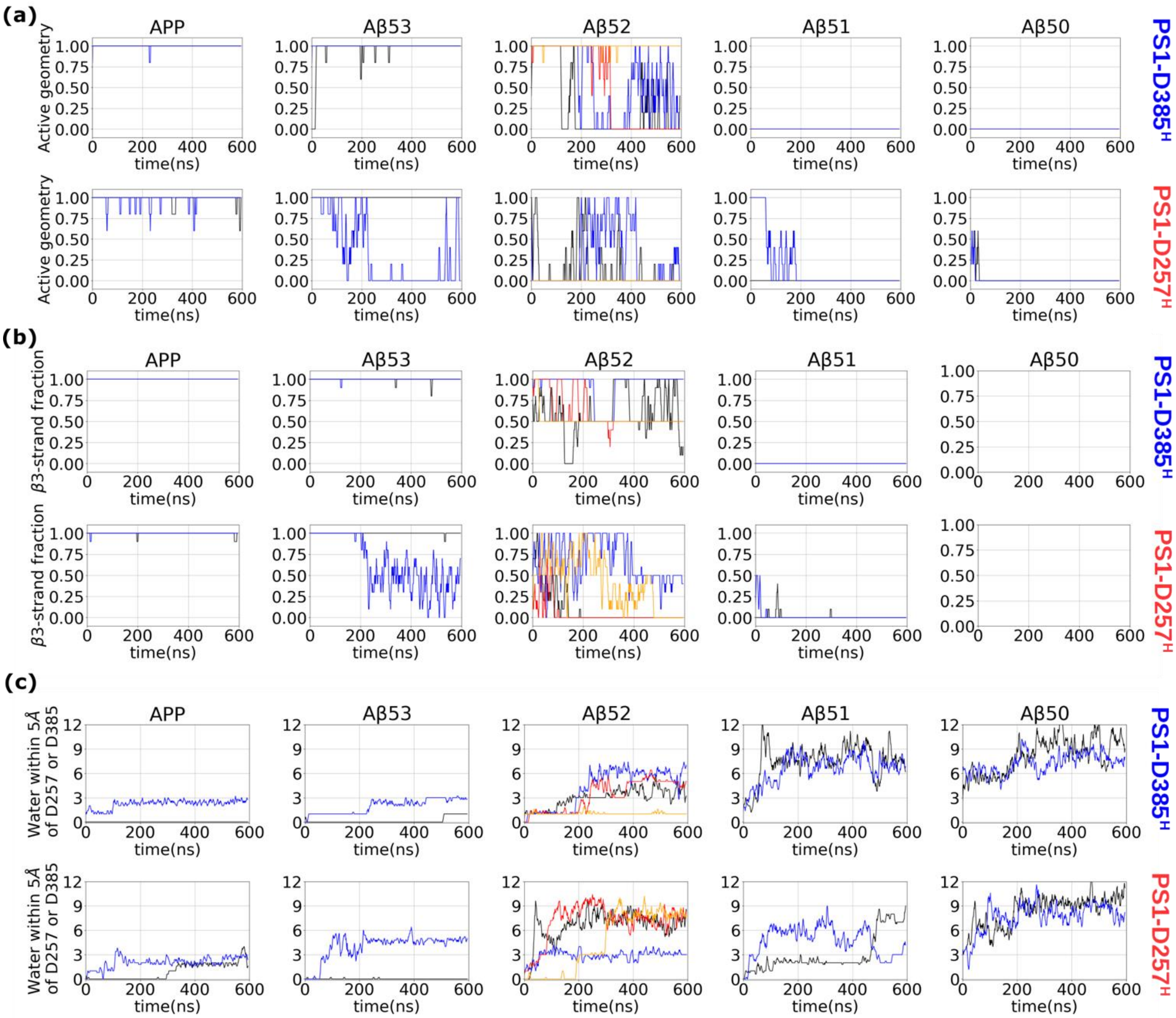

(a) Formation of the active geometry. **(b)** β3-strand formation fraction. **(c)** Number of water molecules within 5Å of D257 or D385. β3-strand formation fraction is not applicable for Aβ50. Data points with substrate binding to γ-secretase-PS1-D385^{H} are shown in the upper row and lower row when binding γ-secretase-PS1-D275^{H}. Data points are rolling-averaged over 5ns. Each color represents an individual simulation with randomly assigned initial velocities. Individual runs are color coded in black (run1), blue (run2), red (run3), and orange (run4).

Supplementary Figure S10: β3-Strand association in the unrestrained MD simulations and its correlation with the Cα-Cα distance between L418 of PS1 and substrate M51.

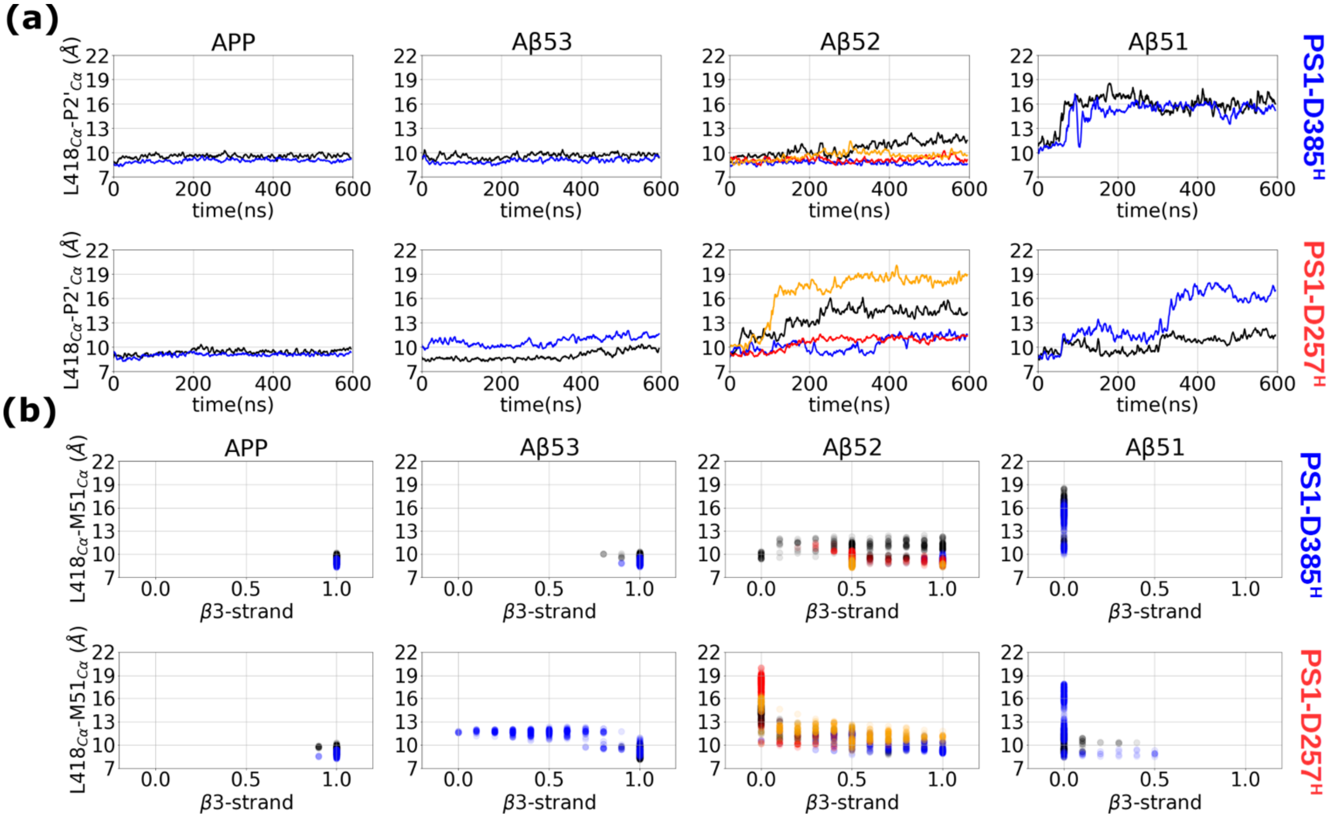

(a) The Cα-Cα distance between L418 of PS1 and substrate M51 in each MD run. **(b)** Correlation between the β3-strand formation and the Cα-Cα distance between L418 of PS1 and substrate M51 in each MD run. Data points with substrate binding to γ-secretase-PS1-D385H are shown in the upper row and lower row when binding γ-secretase-PS1-D275H. Data points are rolling-averaged over 5ns. Each color represents an individual simulation with randomly assigned initial velocities. Individual runs are color coded in black (run1), blue (run2), red (run3), and orange (run4).

Supplementary Figure S11: Hamiltonian Replica exchange MD along the β3-strand association pathway in APP and its truncated variants binding to γ-secretase-PS1-D257[H].

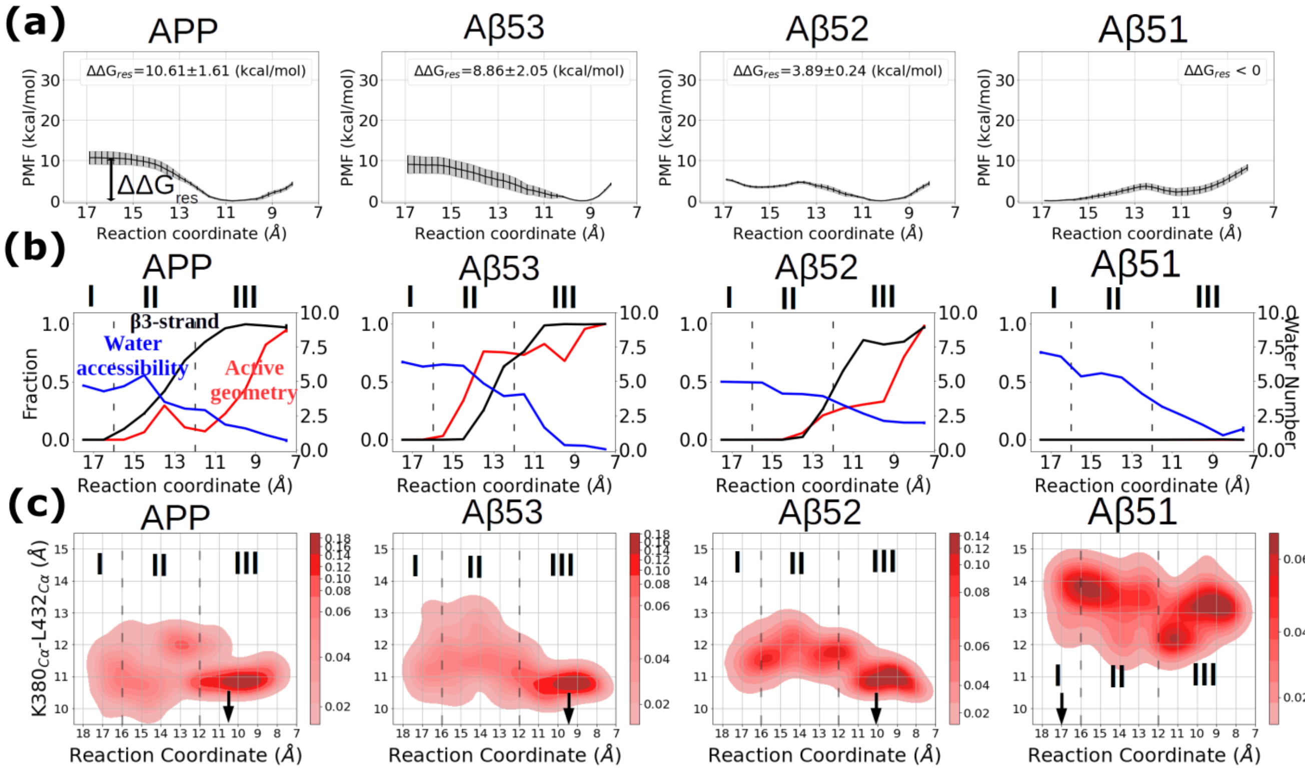

(**a**) Potential-of-mean-force (PMF) profiles calculated with the HREUS method along the substrate β3-strand association reaction of APP and its truncated substrates. **(b)** Fraction of sampled states that form an active site geometry compatible with cleavage (red), β3-strand (black), and the number of water molecules around the catalytic center (blue), along the sampling pathway. **(c)** The change in the gap width between L432 and β2, indicated by the Cα-Cα distance between L432 and K380, along the sampling pathway. The black arrows point to the RC that corresponds to the PMF minimum. Three regimes are distinguished by the transparent dashed lines with regime I corresponding to the dissociated regime, regime II the transition regime, and regime III forming the associated regime. Error bars in **(a)** show the standard deviation of the free energy in each HREUS simulation (n = 16). Error bars in **(b)** show the standard error of the features in each HREUS simulation (n ≥ 15).

Supplementary Figure S12: Decomposition of the binding enthalpy between substrate P1'-P3' and PS1 along the β3-strand association pathway in APP and its truncated variants calculated with MMPBSA.

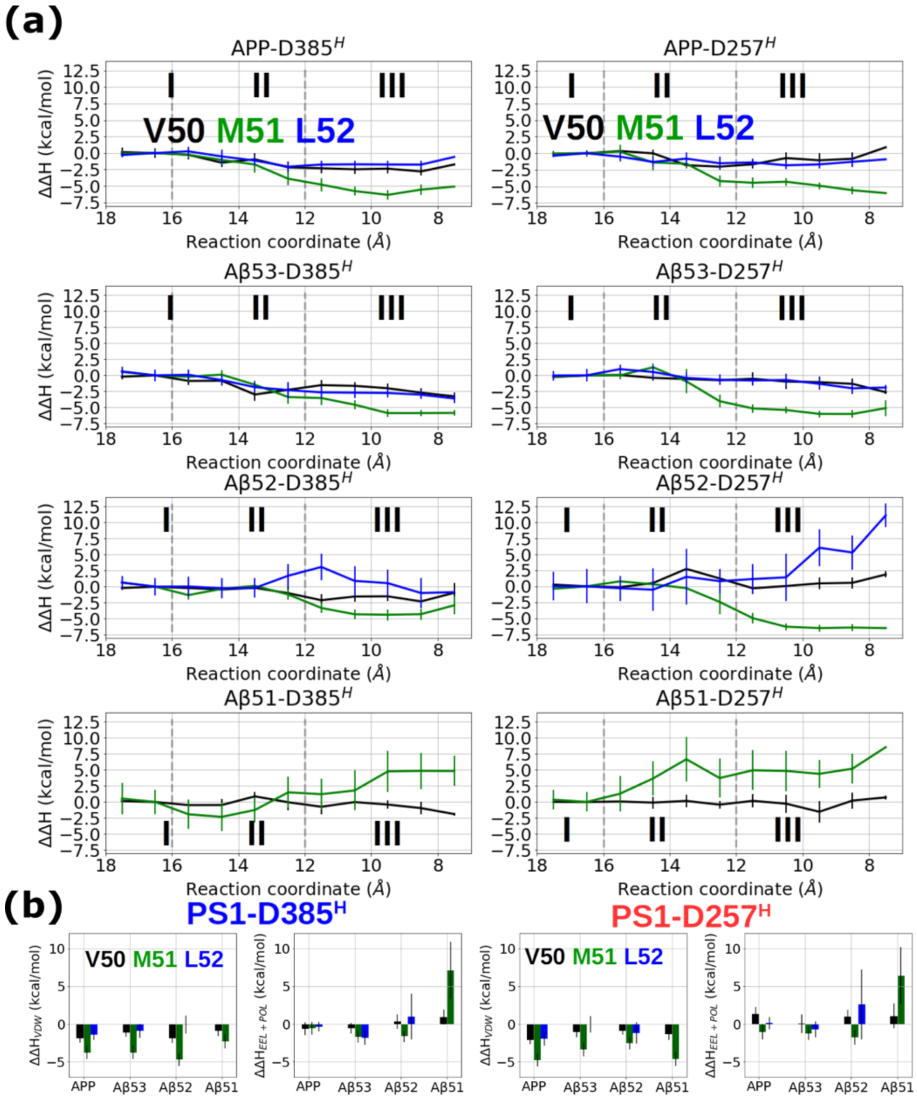

(a) The enthalpy differences from the dissociated state (RC = 16Å) along the RC contributed by V50 (black), M51 (green) and L52 (blue) of APP and its truncated variants binding to D385^H (left) and D257^H (right) PS1. The sample states split by the dashed lines into the dissociated state (regime I), transition state (regime II), and associated state (regime III). **(b)** Energy decomposition of residues' contributions into the Van der Waals (VDW) interaction term and electrostatic/polar-solvation term (EEL+POL) of APP and its truncated variants binding to D257^H (left) and D385^H (right) PS1. Error bars show the standard deviation of the free energy contribution along the RC (n ≥ 15)

Supplementary Figure S13: Hamiltonian Replica exchange MD along the β3-strand association pathway in APP mutants M51P (left) and L52P (right) binding to γ-secretase-PS1-D257^{H}.

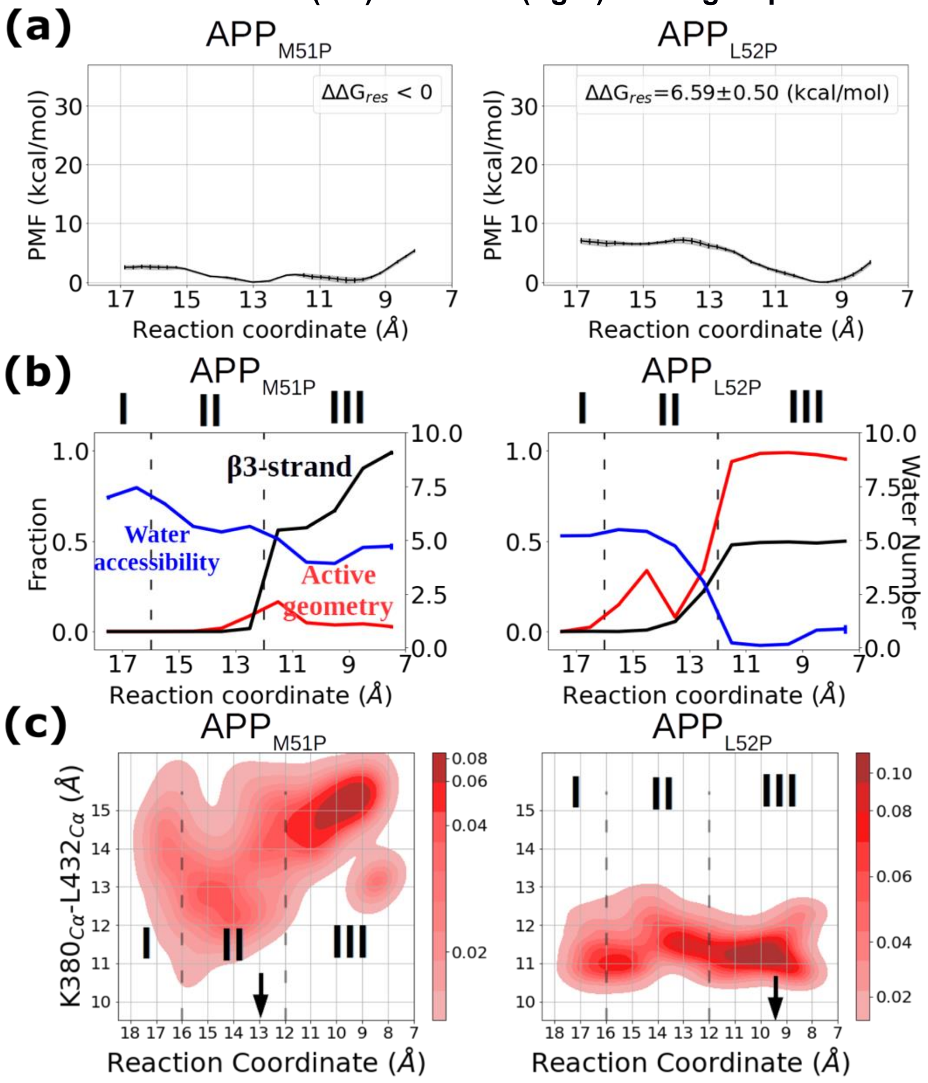

(a) Potential-of-mean-force (PMF) profiles calculated with the HREUS method along the substrate β3-strand association reaction of APP mutants. **(b)** Fraction of sampled states that form an active site geometry compatible with cleavage (red), β3-strand (black), and the number of water molecules around the catalytic center (blue), along the sampling pathway. **(c)** The change in the gap width between L432 and β2, indicated by the Cα-Cα distance between L432 and K380, along the sampling pathway. The black arrows point to the RC that corresponds to the PMF minimum. Three regimes are distinguished by the black dashed lines with regime I corresponding to the dissociated regime, regime II the transition regime, and regime III forming the associated regime. Error bars in **(a)** show the standard deviation of the free energy in each HREUS simulation (n = 16). Error bars in **(b)** show the standard error of the features in each HREUS simulation (n ≥ 15)

Supplementary Figure S14: Decomposition of the binding enthalpy between substrate P1'-P3' and PS1 along the β3-strand association pathway in APP mutants M51P and L52P calculated with MMPBSA.

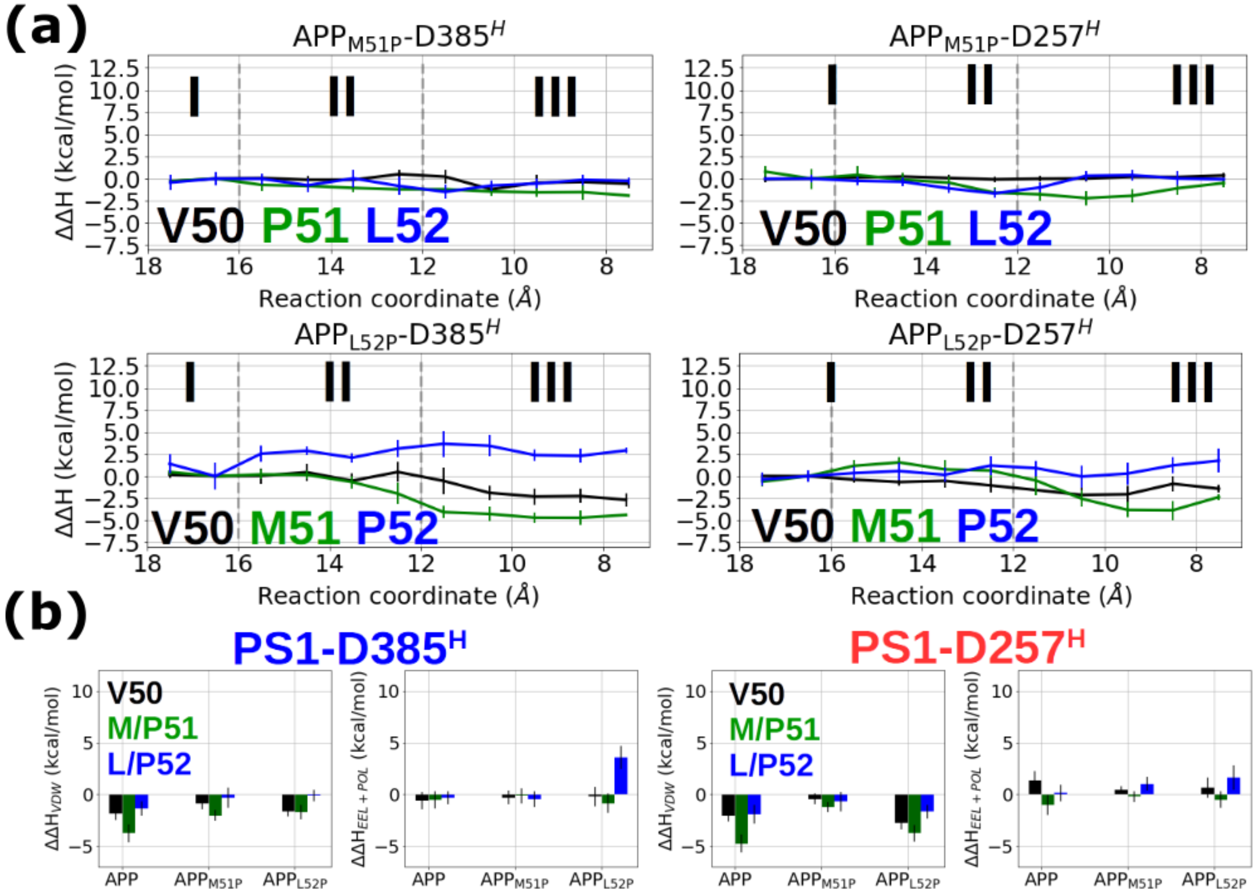

(a) The enthalpy differences from the dissociated state (RC = 16Å) along the RC contributed by V50 (black), M/P51 (green) and L/P52 (blue) of APP mutants binding to $D385^{H}$ (left) and $D257^{H}$ (right) PS1. The sample states split by the dashed lines into the dissociated state (regime I), transition state (regime II), and associated state (regime III). **(b)** Energy decomposition of residues' contributions into the Van der Waals (VDW) interaction term and electrostatic/polar-solvation term (EEL+POL) of APP and its mutants binding to $D257^{H}$ (left) and $D385^{H}$ (right) PS1. Error bars show the standard deviation of the free energy contribution along the RC ($n \geq 15$)

Supplementary Figure S15: Effect of the mutations M51P and L52P on the AICD product species.

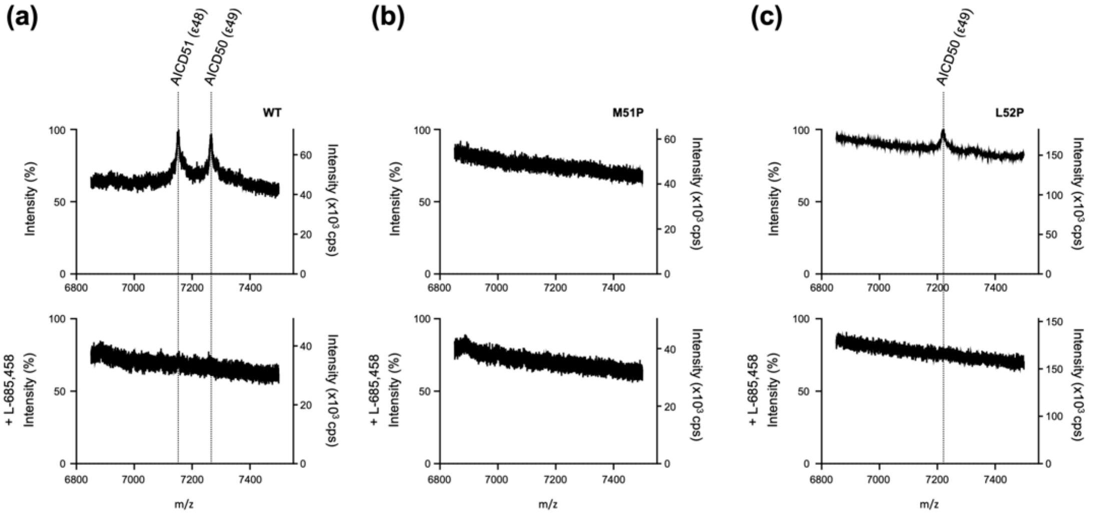

WT		
AICD[#]	Calculated mass [Da]	Observed mass [Da]
AICD51 (ε48)	7234.10	7265.97[1]
AICD50 (ε49)	7120.94	7151.64[1]

M51P		
AICD[#]	Calculated mass [Da]	Observed mass [Da]
AICD51 (ε48)	7200.02	-
AICD50 (ε49)	7086.86	-

L52P		
AICD[#]	Calculated mass [Da]	Observed mass [Da]
AICD51 (ε48)	7218.07	7220.87
AICD50 (ε49)	7104.89	-

MALDI-TOF mass spectrometry analysis species of generated AICD species generated from WT **(a)**, M51P **(b)** and L52P **(c)** mutant C100-His$_6$ substrates. The spectra (upper panel) are shown with their corresponding inhibitor control (middle panel) and the observed and calculated masses of the AICD species (lower panel). The intensity of the highest peak was set to 100%. [#]All AICD species contain a C-terminal hexa-histidine tag which is fused to the C99 C-terminus via a GSRS linker. [1]observed mass corresponds to AICD with an oxidized methionine.

Supplementary Figure S16: The effect of M51F mutant in the associated state (RC=9.2Å).

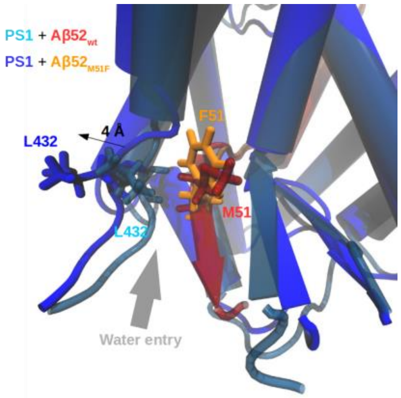

The overlap of snapshot shows the conformations of PS1 when binding to WT Aβ52 (light blue and red) and when binding to the M51F mutant (dark blue and orange). The large Phe sidechain of the M51F mutant causes a 4Å deviation of PS1 L432 (measured by the Cα atoms) and creates an entry for water access to the active site.

Supplementary Figure S17: Hamiltonian Replica exchange MD along the β3-strand association pathway in Aβ52 mutants V50F (left) and M51F (right) binding to γ-secretase-PS1-D257^{H}.

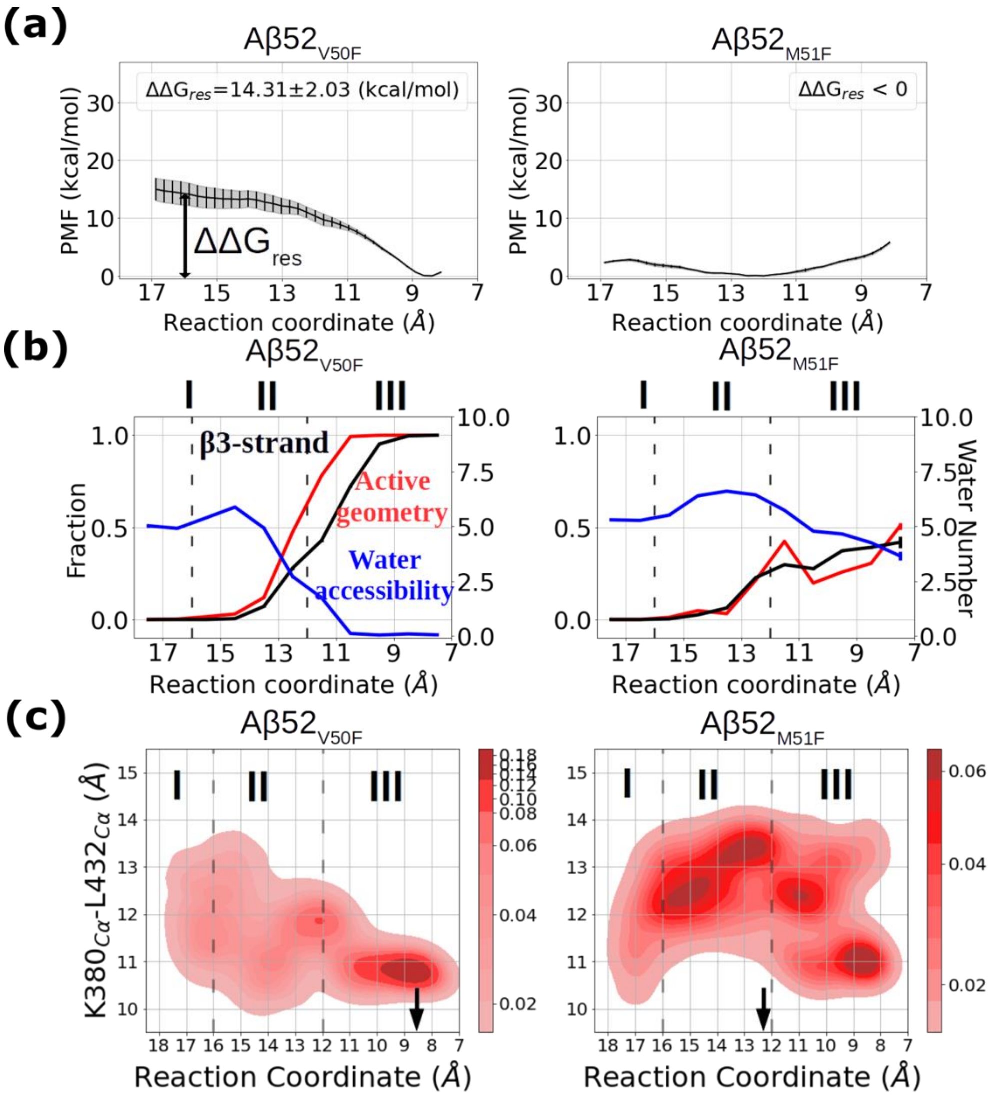

(a) Potential-of-mean-force (PMF) profiles calculated with the HREUS method along the substrate β3-strand association reaction of Aβ52 mutants V50F (left) and M51F (right). **(b)** Fraction of sampled states that form an active site geometry compatible with cleavage (red), β3-strand (black), and the number of water molecules around the catalytic center (blue), along the sampling pathway. **(c)** The change in the gap width between L432 and β2, indicated by the Cα-Cα distance between L432 and K380, along the sampling pathway. The black arrows point to the RC that corresponds to the PMF minimum. Three regimes are distinguished by the transparent dashed lines with regime I corresponding to the dissociated regime, regime II the transition regime, and regime III forming the associated regime. Error bars in **(a)** show the standard deviation of the free energy in each HREUS simulation (n = 16). Error bars in **(b)** show the standard error of the features in each HREUS simulation ($n \geq 15$).

Supplementary Figure S18: Decomposition of the binding enthalpy between substrate P1'-P3' and PS1 along the β3-strand association pathway in Aβ52 mutants V50F and M51F calculated with MMPBSA.

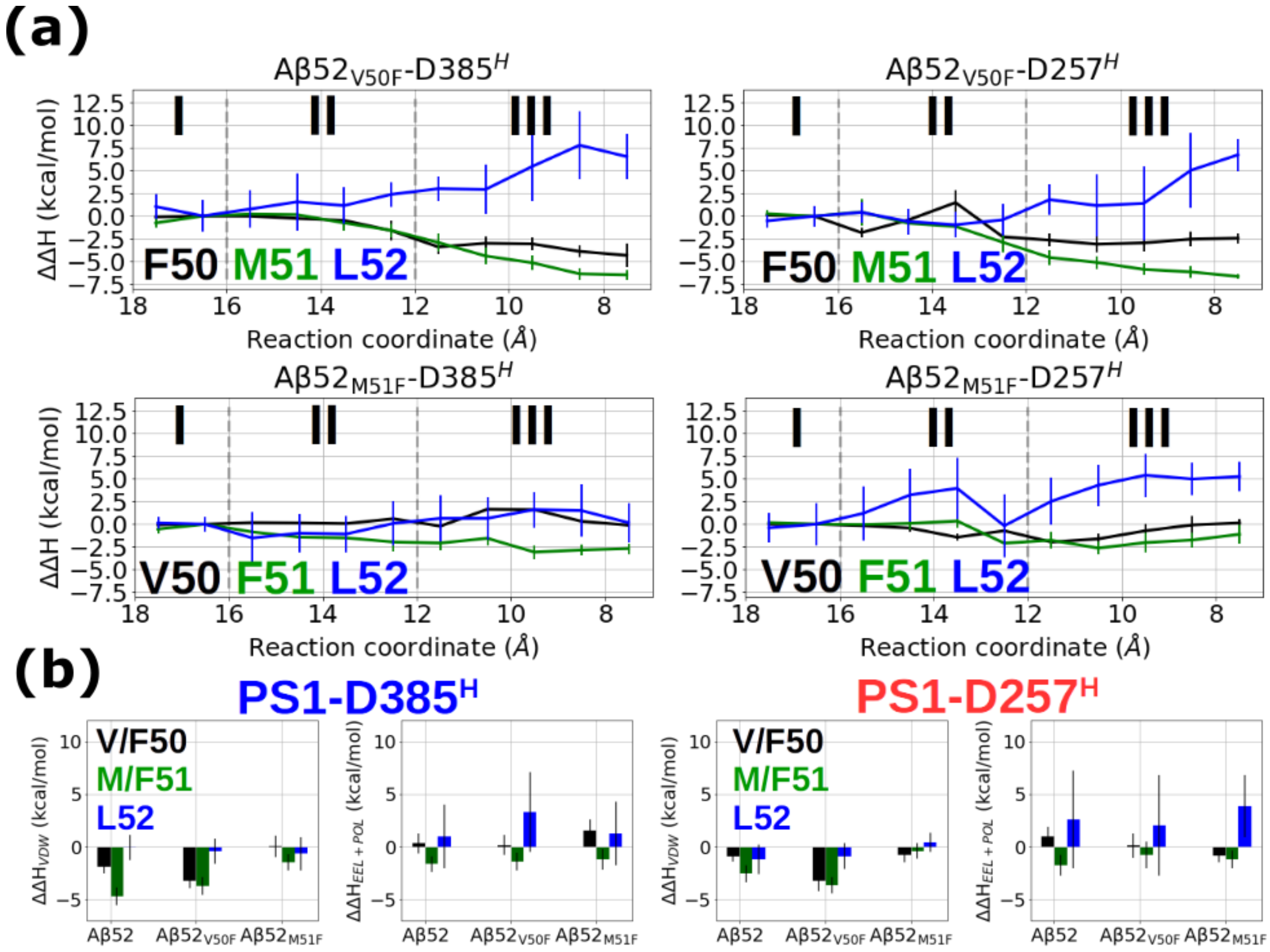

(a) The enthalpy differences from the dissociated state (RC = 16Å) along the RC contributed by V/F50 (black), M/F51 (green) and L52 (blue) of Aβ52 mutants binding to $D385^H$ (left) and $D257^H$ (right) PS1. The sample states split by the dashed lines into the dissociated state (regime I), transition state (regime II), and associated state (regime III). **(b)** Energy decomposition of residues' contributions into the Van der Waals (VDW) interaction term and electrostatic/polar-solvation term (EEL+POL) of Aβ52 and its mutants binding to $D257^H$ (left) and $D385^H$ (right) PS1. Error bars show the standard deviation of the free energy contribution along the RC ($n \geq 15$).

Supplementary Figure S19: β-strand fraction of P1' to P4' residues when binding to D385^{H} γ-secretase identified by DSSP in the HREUS simulations.

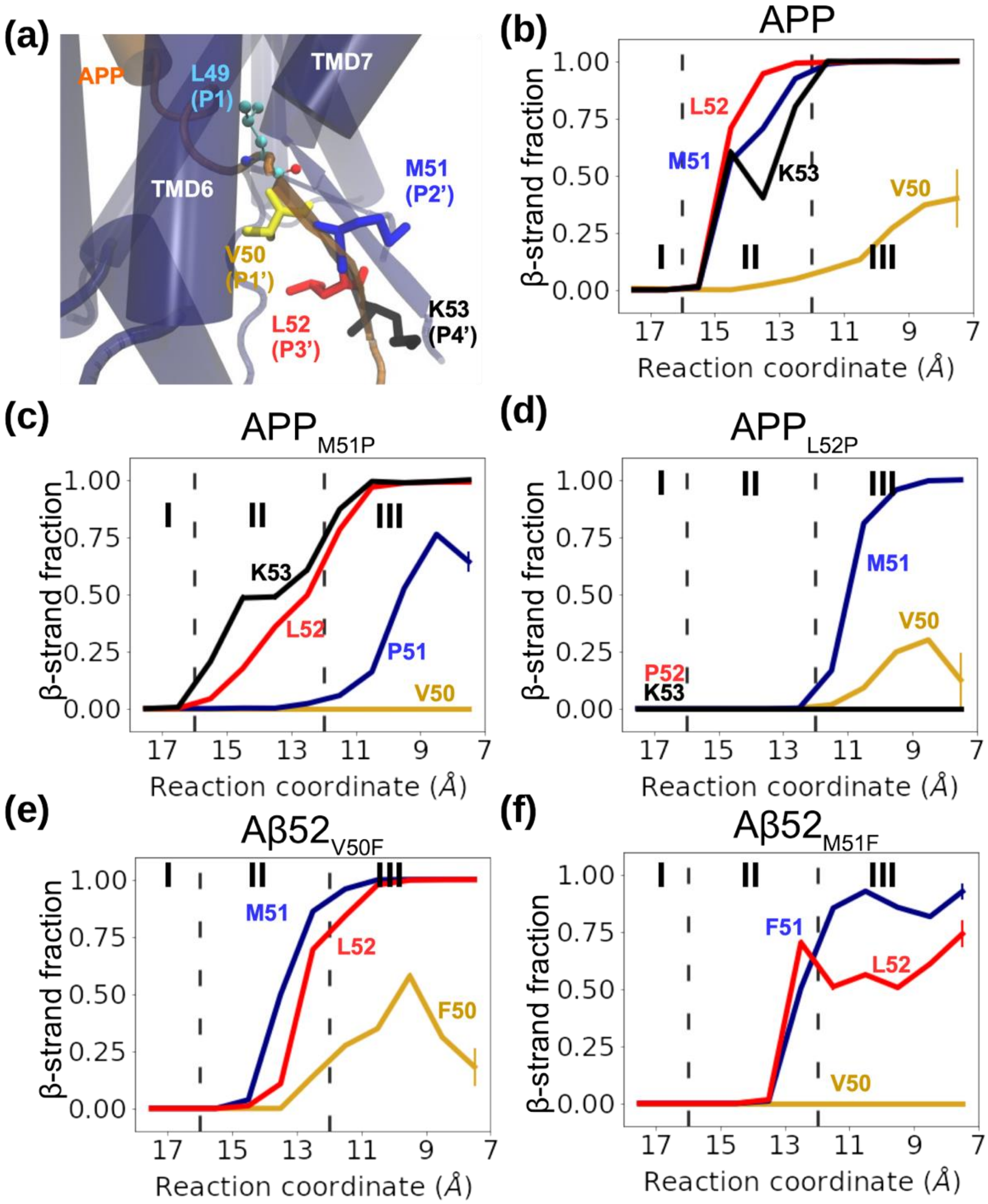

(a) Schematic of the locations of P1 (cyan), P1' (yellow), P2' (blue), P3' (red), and P4' (yellow) of wild type APP (orange) binding to γ-secretase (blue). **(b)**-**(f)** The fraction of frames sampled in the β-strand conformation of P1'-P4' of **(b)** wildtype APP, **(c)** APP$_{M51P}$ **(d)** APP$_{L52P}$ **(e)** Aβ52$_{V50F}$, and **(f)** Aβ52$_{M51F}$ in the HREUS simulations. Phase I (left, dissociated state), Phase II (middle, transition state), Phase III (right, associated state) are separated with black dashed lines. Color of curves correspond to the color coding in **(a)**. Error bars show the standard error of the β-strand fraction along the RC ($n \geq 15$).

Supplementary Figure S20: Hypothetical inhibition mechanism through the disruption of the hybrid β-strand.

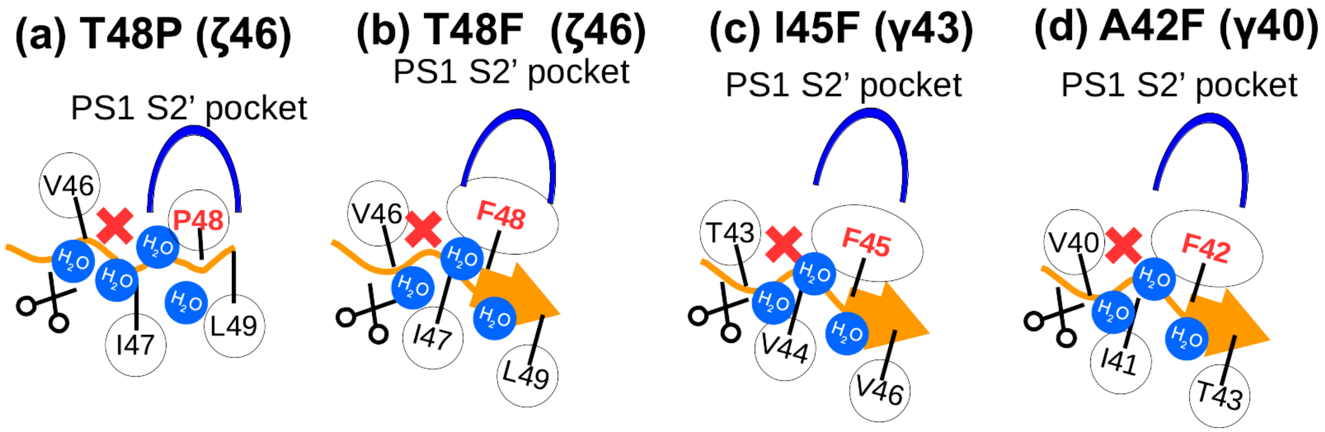

The ζ46 cleavage is impeded by APP mutation **(a)** T48P and **(b)** T48F. The γ43 and γ40 cleavages are impeded by APP mutation **(c)** I45F and **(d)** A42F, respectively.

Supplementary Figure S21: Examples of enzyme-substrate β-sheets formed in other proteases.

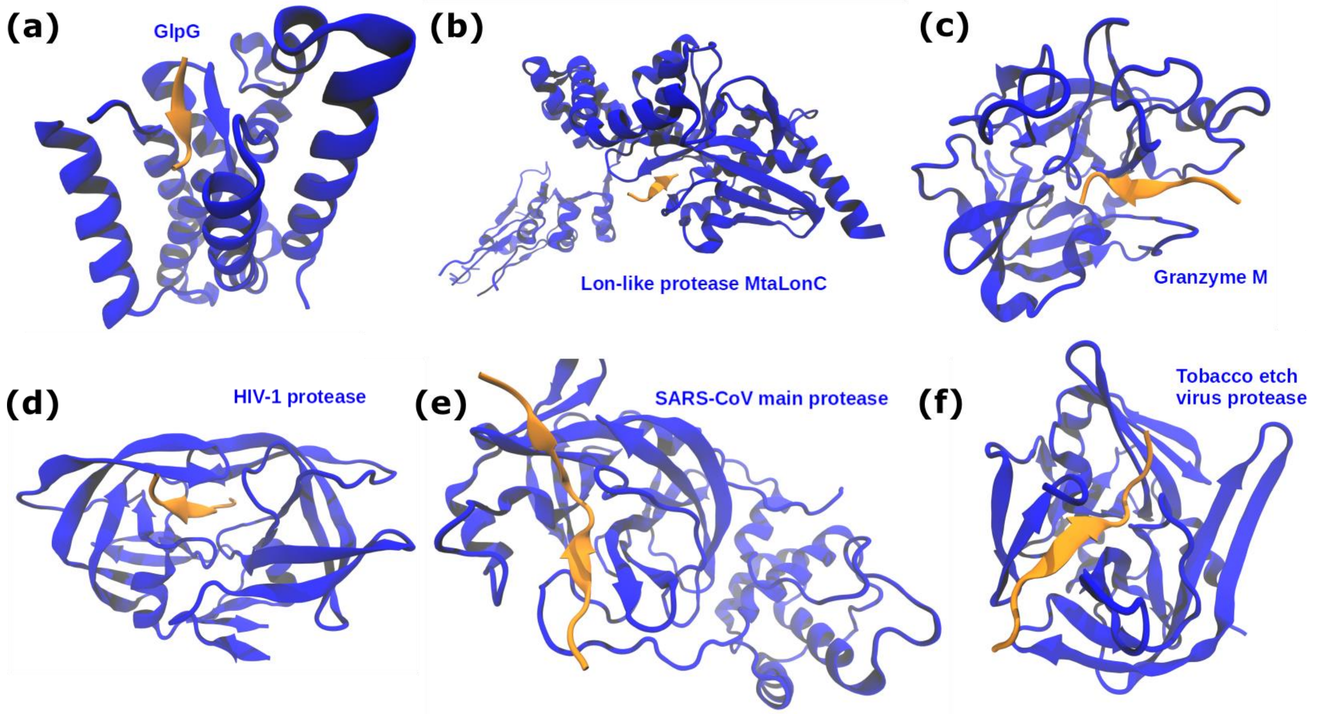

(a) Intramembrane protease GlpG protease (PDBID 6pja) **(b)** Lon-like protease MtaLonC. (PDBID 7eux) **(c)** HIV-1 protease (PDBID 3d3t) **(d)** Granzyme M (PDBID 2zgj) **(e)** SARS-CoV main protease (PDBID 2q6g) **(f)** Tobacco Etch Virus Protease (PDBID 1lvb). The enzymes are colored in blue, and the substrates are colored in orange.

Supplementary Figure S22: Residue-wise root-mean-square fluctuation (RMSF) plot of PS1.

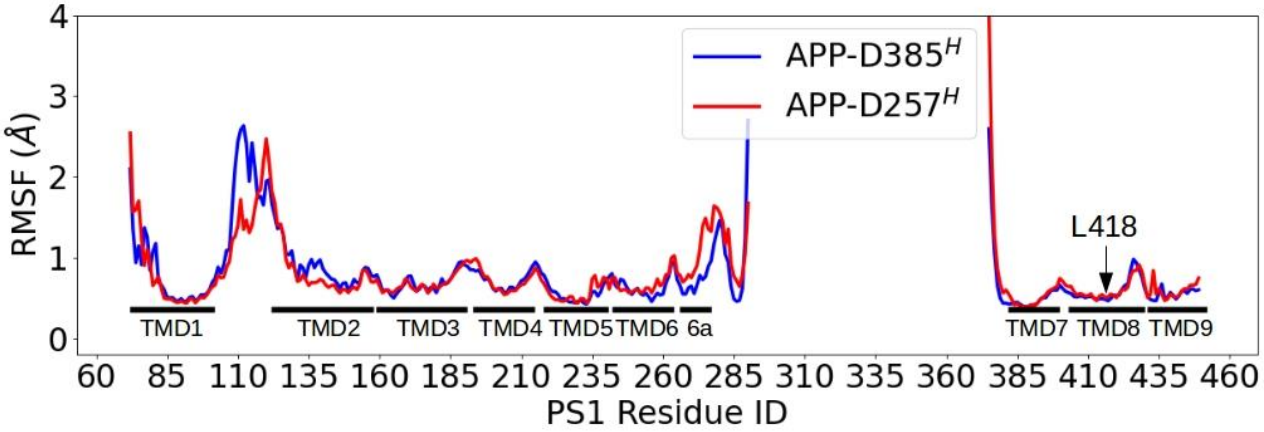

Both APP-bound γ-secretase-PS1-D385^H (blue) and γ-secretase-PS1-D257^H (red) show low fluctuation at residue L418.

Supplementary Figure S23: The sampled RC distance during the 60 ns H-REMD simulation.

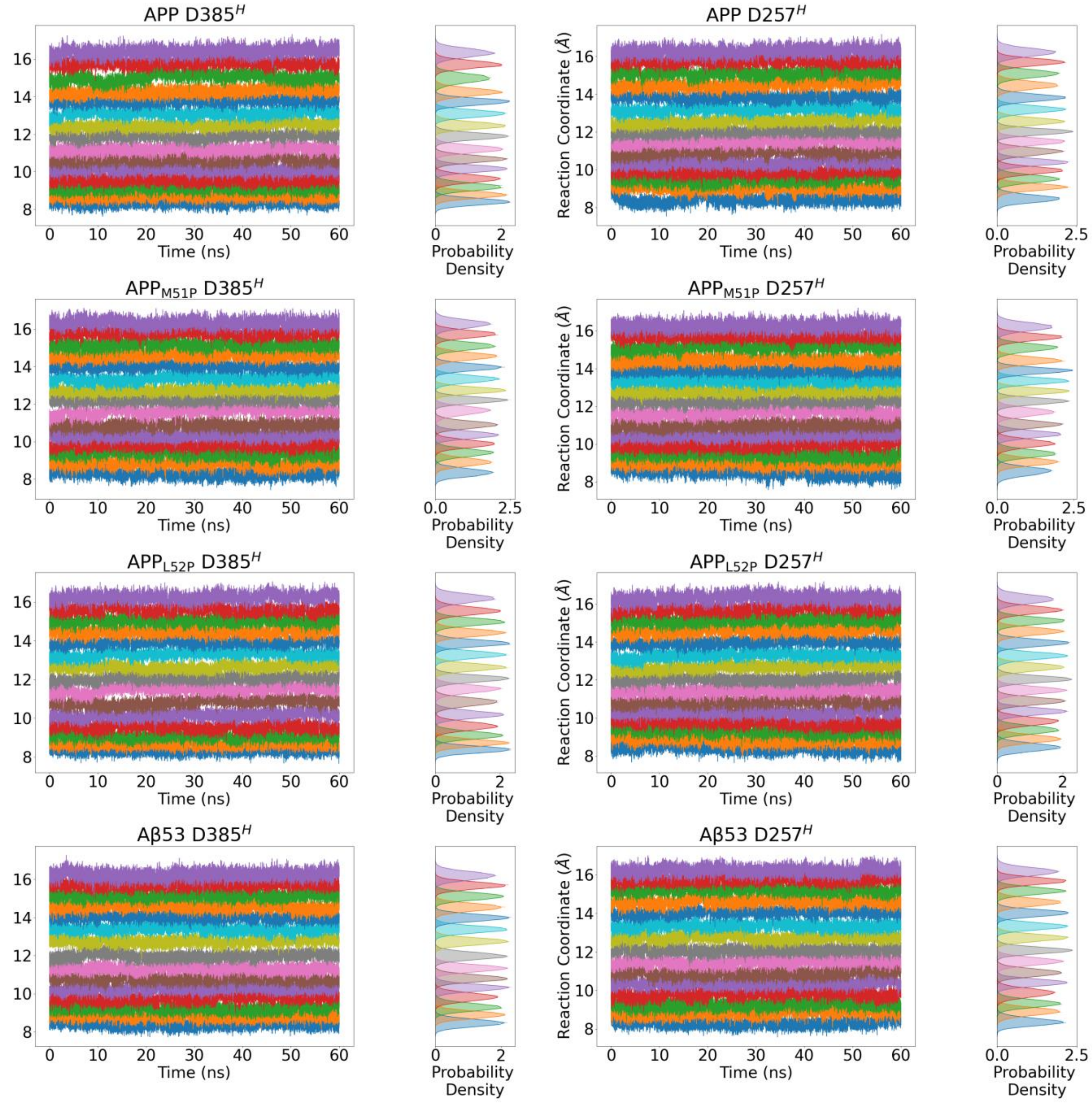

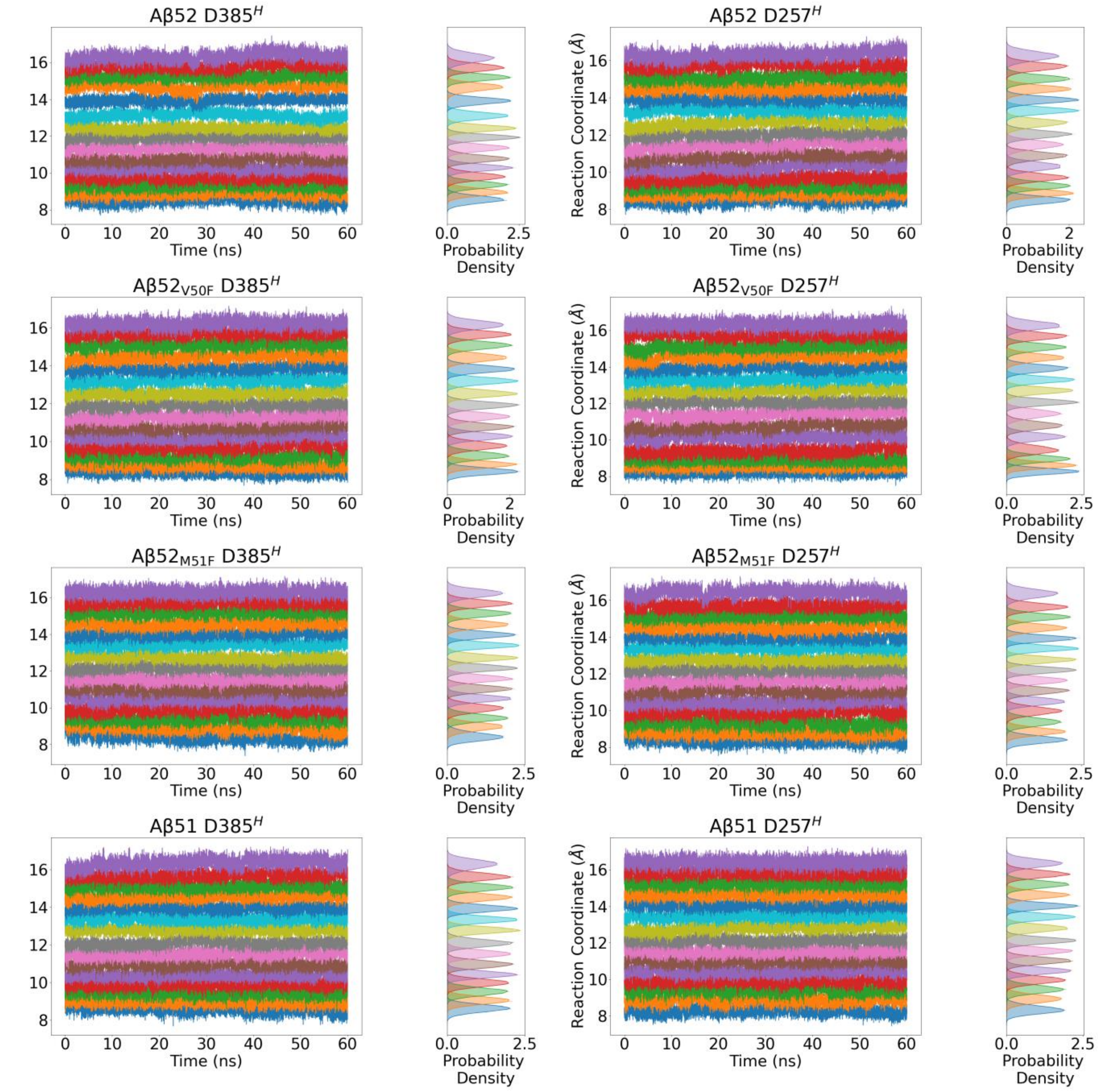

Time plot of the measured RC values in APP, APP$_{M51P}$, APP$_{L52P}$, Aβ53, Aβ52, Aβ52$_{V50F}$, Aβ52$_{M51F}$, and Aβ51 binding to γ-secretase-PS1-D385^{H} and γ-secretase-PS1-D257^{H}.

Supplementary Figure S24: Convergence of PMF profiles along the H-REMD protocol.

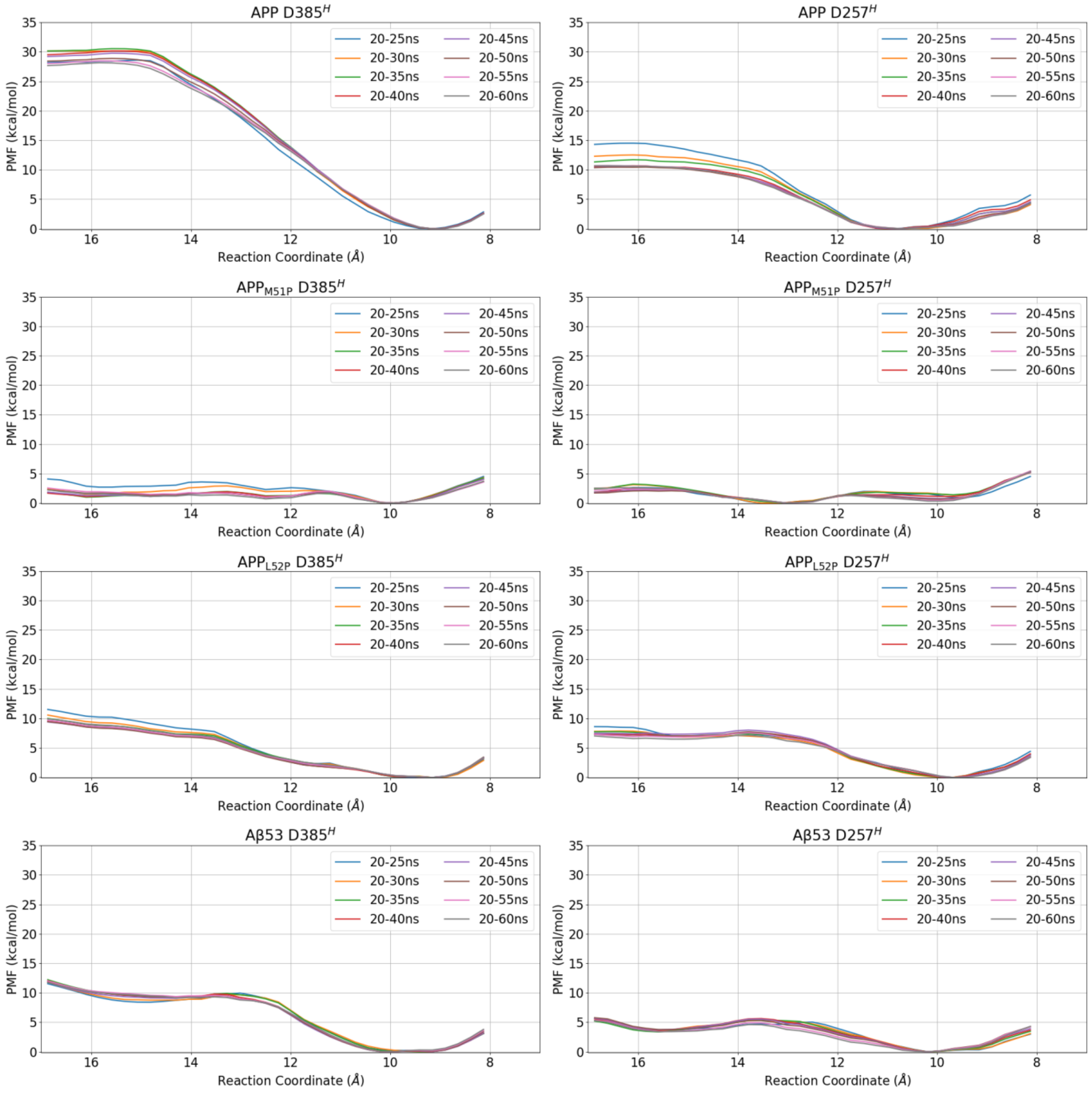

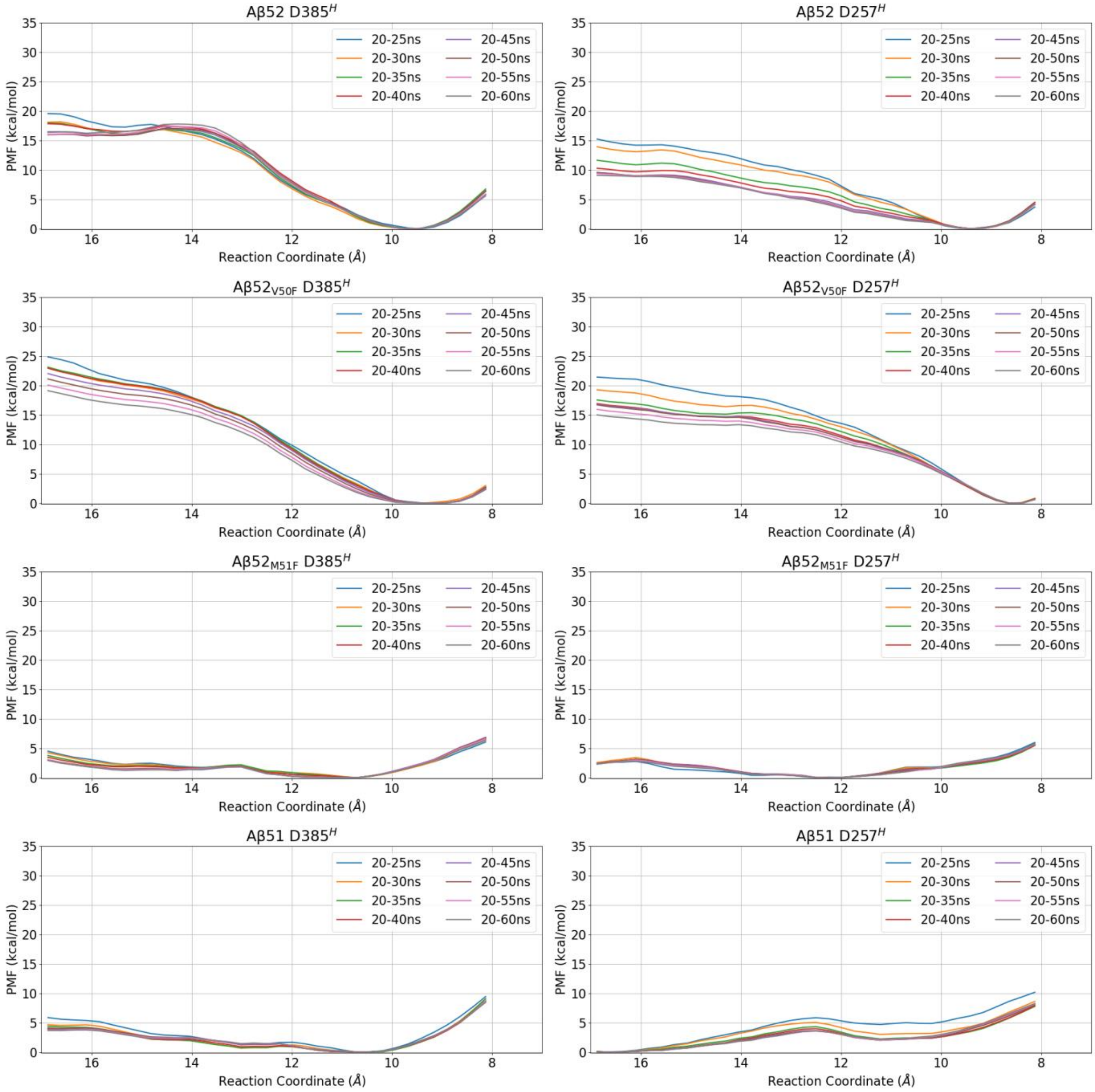

The calculated PMF profiles in the APP$_{M51P}$, APP$_{L52P}$, Aβ53, Aβ52, Aβ52$_{V50F}$, Aβ52$_{M51F}$, and Aβ51 H-REMD simulations binding to γ-secretase-PS1-D385^{H} and γ-secretase-PS1-D257^{H}.

Figure S25 The uncropped blot of the immunoblotting experiments.

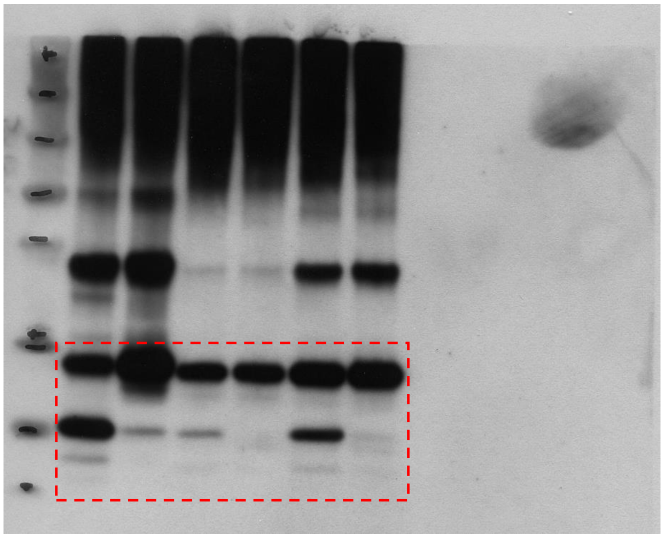

From left to right: $C100_{WT}$-His_6, $C100_{WT}$-His_6 + GSI, $C100_{M51P}$-His_6, $C100_{M51P}$-His_6 +GSI, $C100_{WT}$-His_6, $C100_{WT}$-His_6 +GSI. The region shown in figure 4f is highlighted with the red dashed box.
